# HUSBAND’S EMOTIONAL SUPPORT PROVISION TO ADULT CHILDREN AND WIFE’S MARITAL SATISFACTION IN LATER LIFE

**DOI:** 10.1093/geroni/igz038.2478

**Published:** 2019-11-08

**Authors:** Heejeong Choi, Jaeeon Yoo

**Affiliations:** 1 Sungkyunkwan University, Sungkyunkwan University, Seoul, Korea, Republic of; 2 Korea Institute for Health & Social Affairs, Korea Institute for Health & Social Affairs, Sejong, Korea, Republic of

## Abstract

The current study examined the association between intergenerational support exchange and marital satisfaction among older Korean couples. Prior work has not paid due attention to the fact that older parents and adult children often exchange various types of support in the context of marital relationships, and that provision or receipt of support could influence their marital relationships (see Lee, Zarit, Rovine, Biritt, & Fingerman, 2010; Polenick, Birditt, & Zarit, 2018, for exceptions). Using the 2008 Actual Living Condition of the Elderly and Welfare Need Survey (ALCEWNS), a nationally representative survey of community-dwelling adults 60 years and older, we evaluated the links between marital satisfaction and each spouse’s reports of emotional and instrumental support provided to or received from adult children. For analyses, a series of actor-partner interdependence models were estimated. Findings revealed that wives’ marital satisfaction was associated with their husband’s exchange of emotional support with adult children. By contrast, husbands’ marital satisfaction was unaffected by their wife’s emotional support exchange with adult children. More specifically, wives were more satisfied with their marriage when their husband reported providing greater emotional support to adult children than receiving it from adult children. In addition, wives indicated higher marital satisfaction when the couple provided similar levels of emotional support to their children. Provision or receipt of instrumental support had no bearings on marital satisfaction of either spouse. Taken together, our findings highlight how older couples may evaluate their relationship quality in the Korean cultural context.

